# Investigating the relationships between the burden of multiple sensory hypersensitivity symptoms and headache-related disability in patents with migraine

**DOI:** 10.1186/s10194-021-01294-8

**Published:** 2021-07-19

**Authors:** Keisuke Suzuki, Shiho Suzuki, Tomohiko Shiina, Madoka Okamura, Yasuo Haruyama, Muneto Tatsumoto, Koichi Hirata

**Affiliations:** 1 Department of Neurology, Dokkyo Medical University, 880 Kitakobayashi, Mibu, Shimotsuga, Tochigi, 321-0293 Japan; 2 Integrated Research Faculty for Advanced Medical Science, Dokkyo Medical University School of Medicine, Tochigi, Japan; 3 Medical Safety Management Center, Dokkyo Medical University Hospital, Tochigi, Japan

**Keywords:** Migraine, Sensory hypersensitivity symptoms, Headache-related disability

## Abstract

**Objective:**

Sensory hypersensitivities such as photophobia, phonophobia, and osmophobia are common in patients with migraine. We investigated the burden of these multiple sensory hypersensitivities in migraine.

**Methods:**

In this cross-sectional study, 187 consecutive patients with migraine (26 men/161 women; age, 45.9 ± 13.2 years) were included. Sensory hypersensitivity symptoms such as photo−/phono−/osmophobia and accompanying symptoms were determined by neurologists in interviews. The Migraine Disability Assessment (MIDAS) was used to assess headache-related disability. The Kessler Psychological Distress Scale (K6) was also administered.

**Results:**

Photophobia, phonophobia and osmophobia were observed in 75.4%, 76.5% and 55.1% of the patients with migraine, respectively. A significant overlap in sensory hypersensitivities (photo−/phono−/osmophobia) was found; the proportions of patients with 2 and 3 coexisting sensory hypersensitivities were 33.2% and 41.7%, respectively. The MIDAS score was higher in those with 3 sensory hypersensitivity symptoms than in those with 0 to 2 sensory hypersensitivity symptoms. A generalized linear model with ordinal logistic regression analysis revealed that multiple sensory hypersensitivities, younger age, more migraine days per month, and a higher K6 score were significantly related to the higher MIDAS score.

**Conclusion:**

Our study showed that sensory hypersensitivities commonly occur and overlap in patients with migraine and that multiple sensory hypersensitivity symptoms have a significant impact on headache-related disability.

**Supplementary Information:**

The online version contains supplementary material available at 10.1186/s10194-021-01294-8.

## Introduction

Migraine is a chronic neurologic disorder affecting over 1 billion people worldwide and is known to be the leading cause of disability worldwide in people younger than 50 years [1]. Migraine is characterized by moderate to severe headache, nausea/vomiting and hypersensitivity to visual, auditory and olfactory stimuli. Photophobia, phonophobia and osmophobia are common triggers of migraine attacks and are observed in 50–90%, 52–82% and 25–43% of patients with migraine, respectively [2]. These sensory hypersensitivities are implicated in the underlying pathophysiology of migraine and are related to one another. In previous studies, photophobia was a predictor of osmophobia in patients with migraine [3], and migraine patients with cutaneous allodynia had lower sound aversion thresholds [4]. These observations suggest that migraine is related to not only unimodal sensory processing but also multimodal sensory integration [2]. Headache intensity has been significantly correlated with nausea, vomiting, photophobia, phonophobia, and osmophobia in patients with migraine [5]. However, the impact of these multiple sensory hypersensitivity symptoms on headache-related severity has not been well studied. We conducted a cross-sectional study by hypothesizing that the greater the number of sensory sensitivities a patient has, the greater the effect on the degree of headache-related disability.

## Methods

In a single-center, cross-sectional setting, 200 consecutive outpatients with migraine (30 men/170 women; age, 46.2 ± 13.4 years) were initially recruited; those with incomplete data (*n* = 13) were excluded, for a final sample size of 187. The diagnosis of migraine was made by headache specialists according to the International Classification of Headache Disorders, 3rd edition [6]. Patients with organic brain disease other than migraine were excluded by brain magnetic resonance imaging. This study was approved by the institutional review boards of the Dokkyo Medical University Hospital. All participants provided written informed consent to participate in the study.

Duration of illness, habits (smoking, alcohol consumption, and caffeine consumption), the use of acute and chronic medications for migraine, and aura status were obtained based on clinical medical records. The patients were asked about accompanying symptoms and the presence of photo−/phono−/osmophobia and allodynia during both ictal and interictal phases in face-to-face interviews with headache specialists. The overlap of sensory hypersensitivity symptoms was also determined through face-to-face interviews. The Migraine Disability Assessment (MIDAS) [7] was used to assess disability related to headache. Psychological distress during the past 30 days was evaluated by the Kessler Psychological Distress Scale (K6) [8].

### Statistical analysis

Patients with migraine were classified into four groups according to the number of sensory hypersensitivity symptoms (photophobia, phonophobia, and osmophobia) they presented. Normality was assessed by the Shapiro-Wilk test, and the MIDAS score was found to be non-normally distributed (*p* < 0.001). Therefore, the Kruskal-Wallis test followed by Bonferroni’s multiple comparison test was employed to compare the MIDAS scores of the four groups. A generalized linear model with ordinal logistic regression analysis was used to determine the relationship between MIDAS scores and other factors. Considering the sample size, variables with a *p*-value of less than 0.1 in the univariate analysis were entered into the multivariable analysis. Two-tailed *p* values < 0.05 were considered statistically significant. IBM SPSS Statistics V.26.0 (IBM SPSS, Tokyo, Japan) was used for the statistical analyses.

## Results

**Fig. 1:**
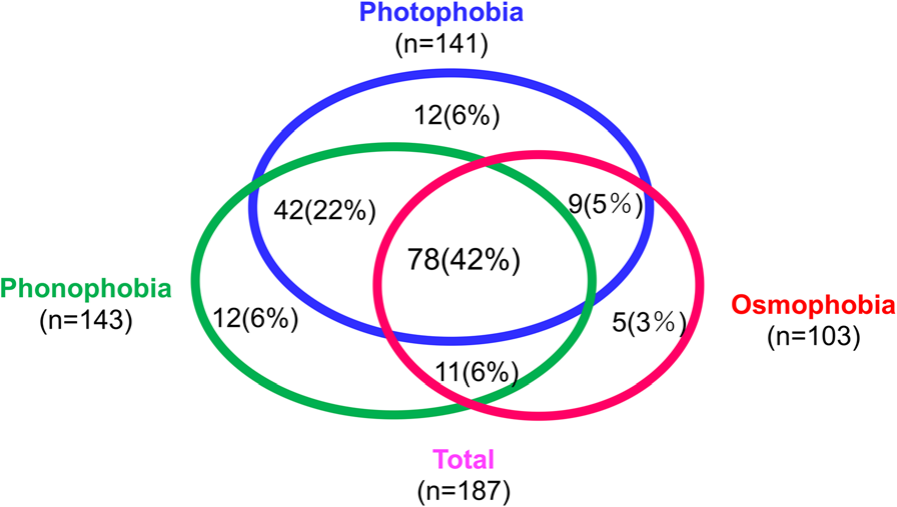
Overlap of sensory hypersensitivities in patients with migraine

**Fig. 2:**
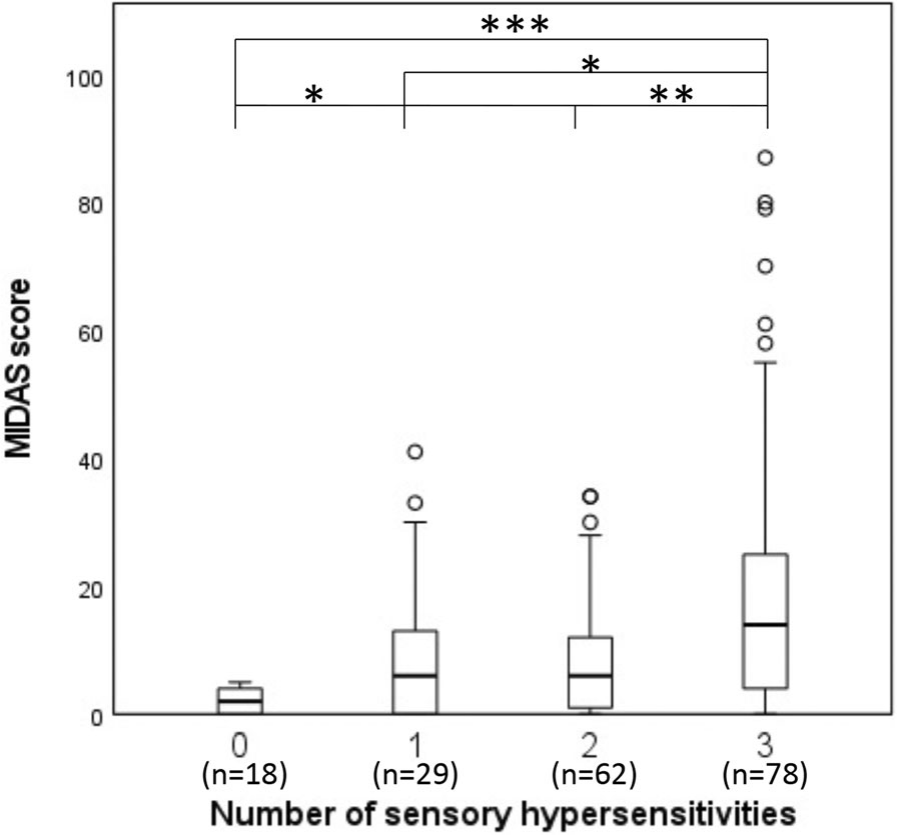
Differences in MIDAS score according to number of sensory hypersensitivities. Box plots show the median, lower and upper quartile, minimum and maximum, and outliers of four sensory hypersensitivities. Each difference among the four groups was analyzed by the Kruskal-Wallis test with Bonferroni’s multiple comparison test. * *p* < 0.05; ** *p* < 0.01; *** *p* < 0.001.

Table 1 shows the demographic data and characteristics of the patients with migraine. The sensory hypersensitivities photophobia, phonophobia and osmophobia were observed in 75.4%, 76.5% and 55.1% of the patients with migraine, respectively. There was a significant overlap in sensory hypersensitivities (photo−/phono−/osmophobia); the proportions of patients with 2 and 3 coexisting sensory hypersensitivities were 33.2% and 41.7%, respectively (Fig. 1). Nausea was reported by 64.2% of patients, and allodynia was observed in 18.2% of patients. In terms of the overlap of sensory hypersensitivity symptoms, the MIDAS score was higher in those with 3 sensory hypersensitivity symptoms than in those with 0 to 2 sensory hypersensitivity symptoms (Fig. 2). The number of sensory hypersensitivity symptoms was not significantly related to the presence or absence of preventive treatment (Supplementary Table 1). The generalized linear model with ordinal logistic regression analysis revealed that multiple sensory hypersensitivities, younger age, more migraine days per month and a higher K6 score were the clinical factors that contributed to a higher MIDAS score (Table 2).

**Table 1:** Characteristics of patients with migraine

	Patients with migraine
n (M/F)	187 (26/161)
Age, mean ± SD, years	45.9 ± 13.2
Diagnosis, n (%)	
Migraine without aura	137 (73.3)
Migraine with aura	50 (26.7)
Chronic migraine, n (%)	8 (4.3)
Migraine days per month, mean ± SD	7.6 ± 7.3
Migraine onset, mean ± SD, years	18.8 ± 8.6
Disease duration, mean ± SD, years	27.1 ± 13.5
Smoking, n (%)	
Never	144 (77.0)
Past	29 (15.5)
Current	14 (7.5)
Alcohol intake, n (%)	
Never	99 (52.9)
< 1 day/week	60 (32.1)
1–2 days/week	16 (8.6)
3–5 days/week	5 (2.7)
6–7 days/week	7 (3.7)
Caffeine consumption, n (%)	174 (93.0)
Nausea	120 (64.2)
Allodynia	34 (18.2)
Sensory hypersensitivity, n (%)	
Photophobia	141 (75.4)
Phonophobia	143 (76.5)
Osmophobia	103 (55.1)
K6 score, mean ± SD	5.3 ± 5.0
MIDAS score, mean ± SD	12.3 (15.8)
Acute headache medication use, n (%)	178 (95.2)
Preventive headache medication use, n (%)	88 (47.1)
Comorbidities, n (%)	98 (52.4)
Psychiatric disease, n (%)	13 (7.0)

*MIDAS* Migraine Disability Assessment, *K6* Kessler Psychological Distress Scale

**Table 2:** The relationship between MIDAS scores and related factors (*n* = 187)

	Univariate analysis^a^					Multivariable analysis^b^				
	COR	95% CI			*p* value	AOR	95% CI			*p* value
Male vs female	0.684	0.328	–	1.427	0.312	0.726	0.310	–	1.700	0.461
Age, years	0.970	0.952	–	0.989	0.002	0.977	0.956	–	0.999	0.038
Migraine without aura, yes vs no	1.439	0.831	–	2.493	0.194					
Migraine with aura, yes vs no	0.745	0.429	–	1.294	0.297					
Chronic migraine, yes vs no	1.706	0.505	–	5.770	0.390					
Migraine days per month, mean ± SD	1.113	1.070	–	1.158	< 0.001	1.094	1.050	–	1.141	< 0.001
Nausea, yes vs no	2.507	1.475	–	4.261	< 0.001	0.658	0.930	–	2.954	0.086
Allodynia, yes vs no	3.057	1.572	–	5.945	< 0.001	1.977	0.972	–	4.022	0.060
Age at first migraine onset, years	0.960	0.932	–	0.990	0.008	0.992	0.959	–	1.026	0.639
Disease duration, years	0.986	0.968	–	1.005	0.149					
Comorbidities, yes vs no	0.875	0.531	–	1.443	0.601					
Psychiatric disease, yes vs no	3.593	1.352	–	9.549	0.010	1.996	0.675	–	2.867	0.096
Acute headache medication, yes vs no	1.579	0.478	–	5.199	0.455					
Preventive headache medication, yes vs no	1.802	1.084	–	2.994	0.023	1.622	0.918	–	2.867	0.096
Smoking										
Never	Ref.					Ref.				
Past	2.044	0.995	–	4.197	0.051	1.778	0.784	–	4.034	0.169
Current	1.871	0.712	–	4.918	0.204	1.372	0.495	–	3.803	0.543
Alcohol intake, n (%)										
Never	Ref.					Ref.				
< 1 day/week	0.272	0.726	–	2.229	0.401	1.542	0.845	–	2.812	0.158
1–2 days/week	1.303	0.519	–	3.272	0.573	1.527	0.564	–	4.134	0.405
3–5 days/week	1.407	0.300	–	6.599	0.665	1.736	0.398	–	7.569	0.463
6–7 days/week	4.449	1.141	–	17.35	0.032	3.003	0.666	–	13.539	0.152
Caffeine intake, yes/no	0.447	0.164	–	1.221	0.116					
Number of sensory hypersensitivities										
0	Ref.					Ref.				
1	3.089	1.085	–	8.794	0.035	1.921	0.626	–	5.902	0.254
2	3.529	1.424	–	8.749	0.006	2.730	1.030	–	7.239	0.044
3	10.427	4.160	–	26.134	< 0.001	5.974	4.845	–	13.372	0.002
K6, score	1.123	1.070	–	1.181	< 0.001	1.068	1.004	–	1.132	0.038

*COR* crude odds ratio, *AOR* adjusted odds ratio, *95% CI* 95% confidence interval, *MIDAS* Migraine Disability Assessment, *K6* Kessler Psychological Distress Scale

^a^Using a generalized linear model with ordinal logistic regression analysis

^b^Using the variables that had *p* values of less than 0.1 in the univariate analysis, except for sex

## Discussion

In this study, we showed a significant overlap of sensory hypersensitivity symptoms, such as photophobia, phonophobia and osmophobia, in patients with migraine. Patients with 3 coexisting sensory hypersensitivities had higher MIDAS scores than patients with fewer sensory hypersensitivities (0 to 2). In addition, the generalized linear model with ordinal logistic regression analysis showed that the higher the number of hypersensitivity symptoms was, the greater the degree of disability related to headache was. In a study by Kelman et al. [5], the more severe a patient’s photophobia, phonophobia, osmophobia, or nausea was, the greater the intensity of the headache was; the authors suggested that the activated pain pathways might activate pathways involving accompanying symptoms such as nausea, or the pathways mediating pain and accompanying symptoms might both be activated simultaneously by another mechanism. However, the impact of the overlap of these factors was not investigated. In contrast, in 92 patients with migraine, interictal sensory hypersensitivities, calculated as combined scores for auditory and visual hypersensitivities, were related to self-perceived attention difficulties but not to headache-related disabilities [9].

Sensory hypersensitivity to stimuli is most prominent during headache attacks but may also be present during interictal periods [2]. Beyond merely accompanying headache attacks, hypersensitivities to light, sound, and smell have also been reported as premonitory symptoms or triggers before a headache attack [10]. This observation suggests that these sensory hypersensitivity symptoms may reflect abnormal brain activity at the earliest stage of a migraine attack in the absence of pain [11]. In a retrospective large-sample study, migraine patients who had nausea or photo−/osmo−/phonophobia in the premonitory phase had a significantly increased frequency of these symptoms as accompanying symptoms of migraine attacks, supporting the idea that hypersensitivity symptoms could be part of the migraine [12]. Neuroimaging studies also reported that non-pain symptoms of migraine were not merely part of the response to pain and produced relevant functional imaging changes even in the absence of pain [11]. Functional brain imaging studies show that patients with migraine have atypical brain activation in response to nociceptive, olfactory, and visual stimuli as well as atypical functional connectivity involved in sensory-discriminative pain processing, affective emotional processing, cognitive processing, and pain modulation [13]. Additionally, in migraine patients, exposure to odors activates the limbic and rostral pons [14], and increased activation of the visual cortex following visual stimulation has been reported [15]. Neuroimaging and electrophysiological studies have supported sensory hypersensitivity in migraine by observing that migraine patients being exposed to sensory stimuli show increased brain activation, a lack of habituation to repeated stimuli, and increased attention to incoming stimuli [16]. The role of multisensory integration in migraine has been suggested by the observation that one sensory modality mediates the presence or intensity of other sensory domain symptoms, as well as by reports of atypical functional connectivity in the temporal pole region, a multisensory convergence zone associated with the processing of visual, olfactory and auditory stimuli [2]. Thus, involvement of cortical and subcortical brain areas and atypical functional connectivity of these areas may explain the contribution of the presence of multiple hypersensitivity symptoms to headache-related disability in our study.

In our study, psychological distress and number of migraine days per month were the relevant factors that contributed to headache-related disability. Chronic stress can be a major trigger for migraines and can amplify the intensity and frequency of headaches due to a hyperalgesic state related to central sensitization or through the activation of N-methyl-D-aspartate (NMDA) receptors or opioid receptors [17]. Relaxation following stress, prolonged fatigue, and extreme tension have been implicated in stress-triggered headaches [18]. Patients with migraine are 1.4 times more likely to develop stress-related headaches than those with tension-type headaches [19]. Interestingly, the association between stressful events and headache was strongest in headache sufferers with low self-efficacy, and this association weakened as self-efficacy increased [20]. Preventive headache medication was significantly associated with MIDAS scores in the univariate analysis, but the significant difference disappeared in the multivariable analysis. The relationship between the use or non-use of preventive headache treatment and sensory hypersensitivity symptoms was not significant. Additionally, it is not clear from this study whether preventive headache treatment could mediate the reduction of the burden of migraine-related disability by improving hypersensitivities.

Our study has several limitations. First, the study design was cross-sectional, and no healthy controls were included. Second, sensory hypersensitivity symptoms were self-reported by the patients, and the presence or absence of sensory symptoms was not assessed using a cutoff on the frequency or severity scale. We also did not assess the subjective severity and frequency of each sensory hypersensitivity symptom, or its degree of consistency or variability among episodes. Thus, it is not possible to explore the correlation between the intensity of hypersensitivities and the degree of migraine-related disability using only the data from this study. Finally, the study setting of an outpatient headache clinic at a university hospital, to which relatively severe cases are referred, may have influenced the results of the study.

## Conclusion

Our study showed that sensory hypersensitivities commonly occur and overlap in patients with migraine and that multiple sensory hypersensitivity symptoms have significant impacts on headache-related disability. Further studies should collect objective measures of the frequency, severity, and overlap of sensory hypersensitivity symptoms and use headache diaries to track their association with headache attacks. Additionally, the efficacy of preventive treatment against sensory hypersensitivity symptoms needs to be evaluated by prospective studies. A better understanding of the relationship among the presence of multiple sensory hypersensitivity symptoms, the pathophysiology of migraine, and the degree of disease-related disability may enhance treatment opportunities.

## Supplementary Information

**Additional file 1.**

## Data Availability

The data sets from this study are available from the corresponding author upon reasonable request.

## Abbreviations

MIDAS: Migraine Disability Assessment
K6: Kessler Psychological Distress Scale
