# Supplementary material for: Investigating the relationships between the burden of multiple sensory hypersensitivity symptoms and headache-related disability in patents with migraine
Source: J Headache Pain. 2021 Jul 19;22(1):77. doi: 10.1186/s10194-021-01294-8 (PMC8287675; doi:10.1186/s10194-021-01294-8)
Supplement: Supplementary file 1 — Additional file 1. [file 10194_2021_1294_MOESM1_ESM.docx]

**Supplementary Table 1: Relationship between preventive headache treatment and number of sensory hypersensitivity symptoms**

| Number of sensory hypersensitivities, n (%) | No preventive treatment (99) | Preventive treatment (n=88) | P value |
| --- | --- | --- | --- |
| 0 | 9 (9.1) | 9 (10.2) | 0.103 |
| 1 | 16 (16.2) | 13 (14.8) |  |
| 2 | 40 (40.4) | 22 (25.0) |  |
| 3 | 34 (34.3) | 44 (50.0) |  |
